# Prioritization and Evaluation of Depression Candidate Genes by Combining Multidimensional Data Resources

**DOI:** 10.1371/journal.pone.0018696

**Published:** 2011-04-06

**Authors:** Chung-Feng Kao, Yu-Sheng Fang, Zhongming Zhao, Po-Hsiu Kuo

**Affiliations:** 1 Department of Public Health and Institute of Epidemiology and Preventive Medicine, College of Public Health, National Taiwan University, Taipei, Taiwan; 2 Institute of Clinical Medicine, School of Medicine, National Cheng-Kung University, Tainan, Taiwan; 3 Departments of Biomedical Informatics and Psychiatry, Vanderbilt University School of Medicine, Nashville, Tennessee, United States of America; 4 Research Center for Genes, Environment and Human Health, National Taiwan University, Taipei, Taiwan; University of Wuerzburg, Germany

## Abstract

**Background:**

Large scale and individual genetic studies have suggested numerous susceptible genes for depression in the past decade without conclusive results. There is a strong need to review and integrate multi-dimensional data for follow up validation. The present study aimed to apply prioritization procedures to build-up an evidence-based candidate genes dataset for depression.

**Methods:**

Depression candidate genes were collected in human and animal studies across various data resources. Each gene was scored according to its magnitude of evidence related to depression and was multiplied by a source-specific weight to form a combined score measure. All genes were evaluated through a prioritization system to obtain an optimal weight matrix to rank their relative importance with depression using the combined scores. The resulting candidate gene list for depression (DEPgenes) was further evaluated by a genome-wide association (GWA) dataset and microarray gene expression in human tissues.

**Results:**

A total of 5,055 candidate genes (4,850 genes from human and 387 genes from animal studies with 182 being overlapped) were included from seven data sources. Through the prioritization procedures, we identified 169 DEPgenes, which exhibited high chance to be associated with depression in GWA dataset (Wilcoxon rank-sum test, *p* = 0.00005). Additionally, the DEPgenes had a higher percentage to express in human brain or nerve related tissues than non-DEPgenes, supporting the neurotransmitter and neuroplasticity theories in depression.

**Conclusions:**

With comprehensive data collection and curation and an application of integrative approach, we successfully generated DEPgenes through an effective gene prioritization system. The prioritized DEPgenes are promising for future biological experiments or replication efforts to discoverthe underlying molecular mechanisms for depression.

## Introduction

Major depressive disorder (MDD) is a complex disorder with high prevalence and is the fourth leading cause of disease burden worldwide [1]. The lifetime prevalence of depression ranges from 9.2 to19.6% worldwide [2]–[4], and heritability is estimated at approximately 37–43% [5]. Over the last decade, many studies have been devoted to dissecting the genetic influences of depression using a variety of experimental designs and technological approaches, including genomic-wide linkage scans, genetic association studies, and microarray gene expression [6]–[12]. Several hypotheses have been proposed for the biological mechanisms of developing depression based on prior evidence [13]–[16], including *monoamine-deficiency hypothesis*, *hypothalamic-pituitary-cortisol hypothesis* and other possible pathophysiological mechanisms (e.g. neurogenesis, abnormal circadian rhythms). Most recently, genome-wide association (GWA) studies have been applied to search for common susceptible variants and genes in several thousands of samples, in turn generating new hypotheses for the biological mechanisms of depression [7], [9], [10], [17]. Massive amounts of genetic data from numerous studies and sources have been accumulated rapidly. Moreover, combining genetic information in the regulatory pathway takes advantage of additional biological knowledge that is not directly available from traditional genetic studies. Results from each study are influenced by different study designs, analytic strategies, ethnic populations, and sample sizes. Thus, integrating depression genetic data and information from individual studies, literature review, and biological pathways in multiple resources may provide us list of evidence-based candidate genes for future experimental validation. Such effort has recently been shown in the study of other complex diseases but has not been applied to depression yet.

One common statistical method to combine results in several studies is meta-analysis, which usually requires data generated by the same design. Findings from various study designs and data sources made it impractical to combine data directly using rigorous statistical testing. Therefore, an alternative powerful integration strategy is needed to combine genetic data from different study settings and across species. Specifically, in neuropsychiatric genetics, several approaches have been developed and applied to integrate genetic data for schizophrenia and Alzheimer's disease. Ma et al. [18] prioritized genes by combining gene expression and protein-protein interaction data for Alzheimer's disease. Sun et al. [19] integrated multi-source genetic data for schizophrenia by a data integration and weighting framework in which the strength of evidence in different data categories is considered and combined by appropriate weights. This approach can be applied to other complex diseases where multi-dimensional data is available. For some complex traits, efforts have been made to integrate and organize data for better utilizing prior research findings, such as a comprehensive and regularly updated Schizophrenia Gene database (Schizophrenia Research Forum, http://www.szgene.org/), an Ethanol Related Gene Resource (ERGR) [20], and a review on the Human Obesity Gene Map for diabetes [21]. In comparison, the progress of identifying biological mechanisms, drug development, and strategies for effective prevention and intervention in response to depression has been relatively slow [22], [23].

Similar to other psychiatric traits, very few significant variants were found from GWA studies due to small effect size [24] in depression, while many more candidate genes were examined in individual genetic studies with inconclusive results. Additional important genetic findings for depression were also derived from mouse models. In the present study, we applied and modified the approach of Sun et al. [19] to effectively integrate multi-dimensional resources of genetic data in both human and mouse studies. We aimed to build up an evidence-based candidate gene framework for depression and used a gene prioritization system to select a final set of depression genes (DEPgenes). We then evaluated the performance of prioritization of DEPgenes by examining the enrichment of small p-values in DEPgenes using a depression GWA dataset and gene expression pattern in human tissues. Our evaluation suggests that our evidence-based DEPgenes might serve as a useful and promising gene source for investigators to further explore the underlying pathophysiology and biological mechanisms for depression.

## Materials and Methods

### 2.1 Candidate genes collection and scoring system

Genetic data was collected from five data sources in human studies and two in animal studies, including association studies, linkage scans, gene expression (both human and animal studies), literature search (both human and animal studies), and biological regulatory pathways. We described the procedures below.

Candidate genes in association studies were searched via published articles of individual studies and meta-analysis. López-León et al. [25] conducted a meta-analysis for MDD and reviewed 183 genetic association studies prior to June 2007, which reported 125 susceptible genes for depression. Among them, 20 genes had polymorphisms in at least three studies. We searched genetic association studies for depression (including binary MDD diagnosis published after June 2007, and measures of depressive mood by validated scales) from NCBI PubMed database. We then manually reviewed them and obtained information on positive or negative associations. Six depression keywords were used. Other than ‘depressive disorder’ for binary diagnosis, we included five quantitative measures: ‘depression symptoms’, ‘Beck depression inventory’, ‘Hamilton depression rating scale’, ‘center for epidemiologic studies depression scale’, and ‘neuroticism’. As a result, we found 141 publications covering 62 genes, all of which were included in the above 125 susceptible genes list. We noticed that there might have publication bias in collecting association data (e.g. 32.8% genes with positive association results only). To reduce possible impacts of publication bias in the study, we did not use original significance level for genes in association studies; instead, we defined a scoring system ranging from 0–4 in an attempt to account for the lower chance of publishing negative findings. We applied two criteria to assign a score for each gene: the total number of studies conducted for a gene and the proportion of positive results among those studies. It is more likely to have an extreme proportion of positive results when the total number of studies related to the gene is small (an extreme example: only one study conducted for a gene and results showing positive association, resulting in a proportion of positive results equaling 1). Hence, we considered both criteria for scoring so the proportion of positive results would not be largely inflated by non-published negative findings. Each gene was given a score (noted as *S_i_*) based on a cut-off for the combinations of the two criteria (see Supplement Table S1 for scoring). A higher score was assigned to a gene if the total number of studies for that gene was large and the proportion of positive results was high. As a result, we had 125 genes with the assigned scores ranging from 0 to 4.

Recently, Harvey et al. [1] reviewed published linkage studies from years 1995 to 2006 regarding mood disorders, and reported 26 genomic regions that showed strong linkage signals to MDD. In addition, we searched individual genome-wide linkage studies in the NCBI PubMed database that were published before 2010 and were not included in Harvey et al. [1] for traits related to affection, including ‘depressive disorder’, ‘bipolar disorder’ and ‘neuroticism’ to obtain extra linkage regions. Three articles [6], [8], [12] were found. Because the resolution in linkage studies was usually low, and it is not easy to define a confidence interval for each linkage peak across many linkage studies, to identify candidate genes (using Ensembl Build 56) in every linkage peak, we arbitrarily defined the boundaries of each selected region by the position of the markers giving the highest logarithm of odds (LOD) scores and extending 10 megabases in both directions. This resulted in a total of 3,628 genes in 33 chromosomal regions. These genes were assigned a score of 1 if their corresponding LOD score ranged between 1 and 2, and the score increased by 1 with an increment of 1 LOD score unit. A score 0 was assigned if the corresponding LOD score was less than 1. Some studies only reported p-values; their −log_10_ *p* values were used in such cases. If both LOD and *p*-values were reported, scores for genes were decided based on the maximum of LOD and −log_10_ *p*. In this data platform, the assigned scores for candidate genes ranged from 0 to 4.6.

To collect gene expression data, we used the Stanley Medical Research Institute online genomics database (SMRIDB). This database collected 12 individual studies using postmortem human brain tissues in 988 array-based expression analyses for depression, schizophrenia and bipolar disorder (https://www.stanleygenomics.org/, November, 2007) [26]. We downloaded the data from the SMRIDB for depression and extracted genes whose *p*-values were less than 0.05; this resulted in 301 genes scored from 0 to 4.6. Scores of these genes were assigned by −log_10_ *p*. To extend the collection of expression data, we additionally searched animal studies of gene expression that examined depression-like behaviors in mice [11]. For these mouse genes, their human homologs were identified by NCBI HomoloGene database (http://www.ncbi.nlm.nih.gov/homologene). Similarly, scores of each gene obtained from animal expression array were assigned by −log_10_ *p*. As a result, we had 252 genes scored from 0 to 5.6.

We also conducted literature searches to identify the relationship between depression and genes, which may not be seen in other data sources described above. It is also possible that genes identified by literature search overlapped with previously identified candidate genes, particularly in data sources of association and microarray studies. Literature searches were conducted using the NCBI PubMed database for the co-occurrence of two entries: a gene name and a depression related keyword to identify their relationship. Since some gene names are identical to meaningful vocabularies (e.g. LARGE, CAT, CLOCK), we used the file “gene2pubmed” downloaded from NCBI-GENE ftp site (ftp://ftp.ncbi.nlm.nih.gov/gene, June, 2010) to identify gene symbols. Six terms (depression, depressive disorder, unipolar disorder, dysthymia, major depression and major depressive disorder) were selected as depression related keywords in human studies. We extracted the unique identifier for a citation (PubMed identifiers, PMIDs) from PubMed. If a gene and a keyword co-occurred in the same reference citation, a hit was identified. Hence, a gene could be scored from 0 (no any hit with depression keywords) to 6 (with all six keywords). In total, 473 genes were scored in human studies. Using the same procedure, literature searches were conducted for mouse studies as well. Six terms related to depressive behaviors in animal models were selected, including forced swim test, tail suspension test, elevate plus maze, novelty induced hypophagia, olfactory bulbectomy and open field test (http://www.natureprotocols.com/2007/12/13/animal_models_for_depressionli.php) according to a review article of Hunsberger et al [27]. Similarly, the human homologs of the mouse genes were identified. As a result, we had 306 genes scored ranging from 0 to 4.

The collection of genes involved in depression-related pathways was more subjective. Based on recent review articles [14], [23], [28] that summarized regulatory pathways in relation to depression using evidence from biological, molecular, and cellular mechanisms, we identified genes that correspond to aforementioned mechanisms, including monoamine-deficiency hypothesis (three pathways), hypothalamic pituitary adrenal axis (four pathways), and other possible pathophysiological mechanisms (five pathways); details please see Supplementary Table S2. Candidate genes were extracted for the 12 pathways via gene-pathway mapping on KEGG (the Kyoto Encyclopedia of Genes and Genomes) database [29], [30]. We assigned a score of 3 to genes that are in the pathways corresponding to the monoamine-deficiency mechanism, a score of 2 for hypothalamic-pituitary-adrenal axis, and a score of 1 for other possible mechanisms. If a gene belongs to more than one mechanism, the greater score was chosen for this gene. We had a total of 827 genes with scores ranging from 1 to 3.

### 2.2 Core genes and GWA dataset

In the candidate genes collection step, we obtained 5,055 genes in total (see Supplementary Table S3). To prioritize these genes according to existing evidence, we used two datasets—a core gene set and a depression GWA dataset—to search for the optimal weights for the seven data sources. Fourteen genes were selected for the core gene set. Six genes (*APOE*, *DRD4*, *GNB3*, *MTHFR*, *SLC6A3* and *SLC6A4*) were based on a meta-analysis for MDD [25], and 8 genes (*BDNF*, *CREB1*, *GRM7*, *HTR1A*, *HTR1B*, *HTR2A*, *MAOA* and *TPH1*) were selected from other review articles for MDD [13], [22], [23]. The GWA data for depression was downloaded through the Genetic Association Information Network (GAIN) (http://www.ncbi.nlm.nih.gov/sites/entrez?db=gap). This MDD GWA data included 1,738 depression cases and 1,802 controls in the Netherlands; a detailed description of this GWA study was provided in Sullivan et al. [10]. A SNP (single nucleotide polymorphism) was assigned to a gene if its location was within the gene or 20kb upstream or downstream of the gene. The smallest *p*-value among the SNPs mapped in a gene was chosen to represent the association signal of the gene. This SNP-gene mapping process resulted in 217,637 SNPs mapped to 15,735 protein-coding genes.

### 2.3 Gene prioritization and evaluation

A gene prioritization framework modified in Sun et al. [19] was applied. A pre-weighting scheme, *preWeight* (0.5 to 1.5), to the seven data sources was originally used to adjust for varying score ranges across data sources (Supplement Table S1). A higher *preWeight* for a platform represents the stronger evidence we subjectively assigned. To check the robustness of the values given in *preWeight*, a second set of *preWeight* (1 for every platform) was also tested. We objectively defined the weighting scheme for data sources (noted as *W_i_*) to weigh their relative magnitude of evidence. Hence, the prioritization system was applied to search for the optimal weight matrix. Briefly, we generated a candidate weight matrix pool consisting of *d^N^* = 8^7^ weight vectors, where *N* represents the number of data sources and *d* = *N*+1 represents possible different weights (i.e. 1 to 8), respectively. The elements in the weight matrix stand for association, linkage, human gene expression, human literature search, regulatory pathway, animal gene expression, and animal literature search, respectively. Each element in a weight vector represents the strength of information/evidence for a platform or data source. Then, a combined score (summation of *preWeight*×*S_i_*×*W_i_*) for each gene could be calculated by summing over the products of the scores and corresponding weights from seven data sources. If a gene shows evidence from multiple data sources, the combined score for such gene would expect to be higher than a gene only with weak evidence in one or two data sources given the optimal *W_i_* has been decided.

In the weight matrix selection step, for each weight matrix, all the 5,055 candidate genes and the core genes were sorted together by their combined scores. Two parameters, *ϕ* (proportion of core genes) and *η* (proportion of candidate genes), were introduced to select weight matrices. Matrices that fulfilled these threshold criteria were retained (see Text S1) for the next evaluation step. The depression GWA data was utilized to evaluate the performance of each retained weight matrix. For each weight matrix, the *p*-values distribution of the top *j* genes (denoted as the prioritized set) and the randomly selected gene set from the GWA data with size *j* (denoted as the random set) were compared using the Wilcoxon rank-sum test. A significant *p*-value (*p*<0.05) represents that the *p*-values distribution in the prioritized set is more significant than in the random set. We generated 1000 random sets in this step for comparisons, and this procedure was repeated 10 times to obtain standard deviation. For every weight matrix, a combined score for each gene could be computed based on the top *j* ranked prioritized gene set. A cutoff value to choose DEPgenes was determined by a clear separation of combined scores distribution between the core genes and the remaining candidate genes. During these prioritization and evaluation steps, a number of weight matrices passed our selection criteria as candidates for the optimal weight matrix.

We applied three approaches to test the robustness of choosing a specific weight matrix as the optimal one to select for DEPgenes (Text S2). First, we selected ten weight matrices that passed selection criteria to evaluate their performance using the GWA dataset. Second, to investigate whether the rank of prioritized genes obtained from each weight matrix was similar, pair-wise comparisons for the ranks of prioritized genes among ten matrices were calculated using Spearman's correlation coefficients. A high correlation on average in these comparisons would demonstrate the effectiveness and robustness of this prioritization approach. Third, we investigated the best matrices obtained from our core gene sets with other two alternative core gene sets for the robustness of our DEPgenes selection: core gene sets based on best expression genes and candidate pathway genes. Finally, we evaluated patterns of gene expression of the DEPgenes and non-disease genes in human tissues. Non-disease genes were used as the reference to compare with the DEPgenes. We retrieved human protein-coding genes and 5,139 disease genes from the GeneCards database (http://www.genecards.org/) and obtained a total of 15,874 non-disease genes. We then compared the gene expression patterns between the DEPgenes and non-disease genes in 49 human tissues that were extracted from the WebGestalt Tissue Expression (http://bioinfo.vanderbilt.edu/webgestalt/) [31] using Wilcoxon signed-rank test. The proportion of the DEPgenes vs. non-disease genes expressed in each tissue was computed.

## Results

A total of 5,055 depression-related candidate genes were obtained from seven data sources, including 4,850 genes in human and 387 genes in animal studies, with only 182 genes (3.6% = 182/5055) overlapping in both species. The percentage of overlapping genes across data sources was low or moderate; it was in a range from 0.3 to 24.8% (Supplementary Table S3), which echoes the challenges we faced to dissect the genetic influences for depression with commonly seen situations of non-replication and inconclusive results. Not surprisingly, there were 12.7% (N = 60) overlapping genes between search by human literature (473 genes identified) and association studies (125 genes identified), indicating a low redundancy between the two data sources (see Table S3).

In the prioritization procedures, too many weight matrices were obtained in the nine sets of parameters (*ϕ* = 0.8, 0.85, 0.9, and *η* = 3, 4, 5%), and we listed only those that met our selection criteria in Table 1. None of weight matrices passed our selection criteria when *ϕ* equals to 0.8 and 0.85. Thirteen weight matrices were reported for *ϕ* = 0.9 (one for *η* = 3% and thirteen for *η* = 4 or 5%) in Table 1. Among them, four matrices, marked in bold, showed better performance than all others with mean ≥950; they also had smaller position *j* and *l*, and they were hence considered as candidates for the optimal weight matrix (definition of *j* and *l* is provided in Text S1). The weight matrix [2,1,1,8,1,1,7] had the highest mean value of 963.9 (i.e. among 1000 comparisons, there were on average 964 times the selected prioritized gene sets had smaller *p*-value distribution than randomly selected gene sets from GWA data). In addition, the prioritized gene sets obtained by this matrix had high proportion to exhibit small *p*-values (<0.05) in the GWA dataset (Supplementary Figure S1). Thus, we selected matrix [2,1,1,8,1,1,7] as our final weight matrix for the seven data sources to calculate combined score for each candidate gene, which equals to (3, 1, 1.5, 4, 1, 1, 3.5) when multiplied the best matrix by *preWeight*. Notably, the weights of three data sources (association studies and literature searches for both human and animal studies) were high, indicating the evidence from association studies and text-mining was more informative than that of the other sources.

**Table 1:** Selection of the optimal weight matrix by core genes and evaluation by the genome-wide association (GWA) *p*-values.

Parameter set^a^		Total number of weight matrices	Number of weight matrices met criteria^b^	Selection by core genes			Selection by GWA *p*-values^f^	
***ϕ*** (%)	*η* (%)			Weight matrix^c^	Position *j* d	Position*_l_* e	Mean	sd
90	3	4,663	1	**[2,1,1,8,1,1,7]**	**150**	**1053**	**966.6**	**5.4**
	4	11,916	13	**[2,1,1,8,1,1,7]**	**150**	**1053**	**963.9**	**4.1**
				[2,1,1,8,1,1,8]	153	738	933.8	5.5
				**[3,1,1,8,1,1,7]**	**153**	**1054**	**952.6**	**7.2**
				[3,1,1,8,1,1,8]	155	738	930.2	9.4
				[4,1,1,8,1,1,7]	157	1054	948.2	7.2
‵				[4,1,1,8,1,1,8]	159	739	922.2	9.3
				[5,1,1,8,1,1,7]	157	1054	944.2	4.5
				[5,1,1,8,1,1,8]	159	739	928.7	6.5
				[6,1,1,8,1,1,7]	159	1054	949.4	8.1
				**[7,1,1,8,1,1,7]**	**159**	**1054**	**952.4**	**7.1**
				**[8,1,1,8,1,1,7]**	**159**	**1054**	**955.3**	**7.5**
				[7,1,1,6,1,3,7]	159	1159	922.3	12.7
				[8,1,1,6,1,3,7]	159	1159	927.4	6.2
	5	20,285	13	**[2,1,1,8,1,1,7]**	**150**	**1053**	**963.0**	**5.4**
				[2,1,1,8,1,1,8]	153	738	931.9	10.5
				**[3,1,1,8,1,1,7]**	**153**	**1054**	**955.2**	**8.4**
				[3,1,1,8,1,1,8]	155	738	931.1	6.5
				[4,1,1,8,1,1,7]	157	1054	942.9	9.6
				[4,1,1,8,1,1,8]	159	739	925.3	7.4
				[5,1,1,8,1,1,7]	157	1054	945.2	9.6
				[5,1,1,8,1,1,8]	159	739	925.0	10.6
				[6,1,1,8,1,1,7]	159	1054	948.9	4.1
				**[7,1,1,8,1,1,7]**	**159**	**1054**	**956.0**	**5.8**
				**[8,1,1,8,1,1,7]**	**159**	**1054**	**951.2**	**7.9**
				[7,1,1,6,1,3,7]	159	1159	922.2	8.9
				[8,1,1,6,1,3,7]	159	1159	921.7	17.6

Note:

a *ϕ* and *η* denote threshold proportion in the core gene set and the candidate gene set.

b Selection criteria: position *j*≤160, position *l*≤1200 and mean≥900. Definition of *j* and *l* is shown in footnote d and e below. The weight matrices with mean 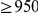 marked in bold.

c Weight matrix is ordered by ω_association_, ω_linkage_, ω_expression_human_, ω_literature_human_, ω_kegg_, ω_expression_rat_, ω_literature_animal_.

d Position *j* represents the position of the *ϕ*-th core gene locates in the *η*-th top ranked candidate genes.

e Position *l* represents the position of the last core gene locates in the ranked candidate genes.

f Mean: total number of random subsets having significant different p-value distribution from the top ranked candidate genes (Wilcoxon rank-sum test, *p*<0.05); sd: standard deviation.

To examine the robustness of optimal weight matrix selection, nine other weight matrices were selected with slightly different weight combinations (also fit criteria of position *j* ≤200, position *l* ≤2500 and mean ≥900). All ten matrices showed a very similar pattern in terms of their *p*-values distribution of derived prioritized gene sets (see Supplementary Figure S1). In addition, ranking of prioritized gene sets generated by the ten matrices were highly correlated with each other (mean correlation coefficients was 0.92), suggesting that the DEPgenes selected for depression by the current gene prioritization system are effective (see Supplementary Table S4). On the contrary, without the procedure of selecting optimal weight matrix (i.e. use [1,1,1,1,1,1,1] matrix), the resulting prioritized gene set had poor performance with low proportion of small *p*-values (i.e. *p*<0.05) in GWA dataset, indicating our weighting scheme for different data sources is strongly recommended. Alternatively, we tested the optimal weight matrices using the best expression and pathway genes as core gene sets to find alternative sets of optimal weight matrices (see Text S3). No any matrix passed our matrix selection criteria using expression core gene set. For pathway core gene set, matrix [6,2,1,8,7,1,8] was identified as the optimal matrix. Information extracted from literature search and association studies is high that was similar to results from original core gene set. There were 85 genes overlapped between the DEPgenes and pathway-DEPgenes; 29 out of 114 pathway-DEPgenes were not included in the original DEPgenes and the average combined score of these 29 genes (9.39) was much lower than the cutoff value of 15. These results revealed comparable findings from different matrices used and our selection of DEPgenes is robust.

**Figure 1:**
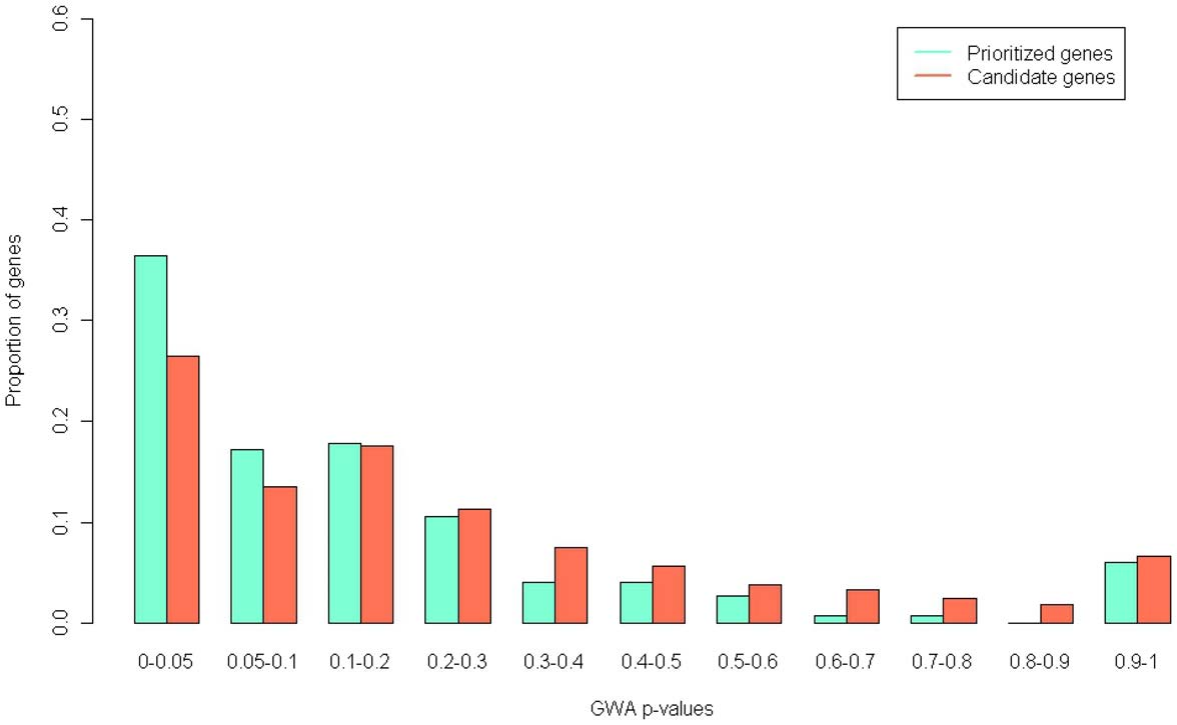
Comparisons of *p*-values distribution using the GAIN GWA-depression data for the 169 prioritized DEPgenes and the remaining candidate genes (N = 4886).

The distributions of combined scores of the 14 core genes and the 5,055 candidate genes differed (see Supplementary Figure S2), and a cutoff value of 15 for combined score was chosen to obtain good discriminability in separating a core gene set from the total candidate genes to select final DEPgenes. A total of 169 genes whose combined scores greater than 15 were selected as DEPgenes (see Table 2). The *p*-values distribution using the GWA dataset for the 169 DEPgenes compared with the 5,055 candidate genes is displayed in Figure 1. The DEPgenes had significantly higher probability (36.4%) to have *p*-values less than 0.05 than the remaining candidate genes (26.5%) using Wilcoxon rank-sum test (*p* = 0.00005).

**Table 2:** The 169 DEPgenes with combined score≥15.

Gene	Combined score	Gene	Combined score	Gene	Combined score	Gene	Combined score	Gene	Combined score
*DBH*	47.74	*TPH2*	28.50	*NOS1*	21.94	*ANXA2*	18.07	*TSNAX*	16.41
*BDNF*	46.52	*CRH*	28.03	*PRKACA*	21.93	*GNB3*	18.04	*TFCP2*	16.39
*SLC6A4*	46.29	*ACCN2*	27.79	*GFAP*	21.87	*ADRB1*	18.03	*CYP2C19*	16.39
*NGFR*	46.04	*ESR1*	27.67	*IL1B*	21.86	*POMC*	18.01	*GRIA1*	16.36
*TNF*	43.61	*ACSL4*	27.58	*HTR2C*	21.72	*NOS3*	18.00	*FMR1*	16.35
*GSK3B*	40.25	*SLC6A3*	27.20	*TH*	21.42	*D2S2944*	18.00	*SLC5A4*	16.32
*CHRNA7*	40.24	*SLC6A2*	26.99	*CYP1A1*	21.33	*DXS7*	18.00	*CC2D1A*	16.32
*GABRA3*	37.55	*ADRA2A*	26.87	*HTR5A*	21.00	*GABRA5*	18.00	*GLI2*	16.30
*CYP2C9*	37.33	*CNR1*	26.64	*SMPD1*	20.97	*LBP*	18.00	*CAT*	16.29
*NTRK3*	37.17	*NQO1*	26.41	*APAF1*	20.79	*M6PR*	18.00	*L1CAM*	16.28
*ADCY7*	36.13	*AVPR1B*	26.30	*TSPO*	20.57	*MAMDC1*	18.00	*CDH17*	16.27
*PDLIM5*	35.99	*TACR1*	26.16	*GNAL*	20.48	*MCP1*	18.00	*DNAJB1*	16.27
*HTR1A*	35.50	*GAD2*	26.12	*GMIP*	20.29	*PDE11A*	18.00	*CD3E*	16.22
*P2RX7*	35.39	*TAAR6*	26.00	*TOR1A*	20.23	*SLC5A7*	18.00	*MTHFR*	16.19
*HTR2A*	35.29	*TPH1*	25.81	*CCKAR*	20.10	*VMAT2*	18.00	*VEGFA*	16.18
*CCL2*	35.24	*HTR3A*	25.77	*CTLA4*	20.00	*ABCB1*	17.89	*DNMT3B*	16.15
*PDE9A*	34.90	*GABRA1*	25.67	*ADCYAP1*	20.00	*PDYN*	17.71	*AKT1*	16.14
*DAOA*	34.00	*ESR2*	25.61	*CYP1A2*	19.89	*SAT1*	17.35	*SERPINE1*	16.13
*NPY*	33.68	*COMT*	25.55	*GNAS*	19.88	*S100B*	17.34	*HSPA12A*	16.13
*GRIN1*	33.44	*PLA2G2A*	25.53	*FKBP5*	19.61	*CD63*	17.14	*S100A10*	16.11
*CHRM2*	33.25	*GAD1*	25.51	*GDNF*	19.50	*GABRA6*	17.12	*CYP2D6*	16.07
*NR3C1*	32.95	*DTNBP1*	25.00	*PLA2G4A*	19.42	*ALDH1A1*	17.03	*GABRA2*	16.03
*DRD4*	32.77	*ACE*	24.14	*ADRA1A*	19.17	*RELN*	17.00	*PTX3*	16.00
*CREB1*	32.54	*XBP1*	23.41	*SLC1A4*	18.88	*ATP6V1B2*	16.87	*C5orf20*	16.00
*DRD3*	32.31	*MAOB*	23.36	*HSPA1A*	18.83	*CAMK2A*	16.68	*GHRL*	16.00
*CRHR2*	31.97	*P2RX4*	23.35	*CACNA1C*	18.52	*BCR*	16.58	*IVNS1ABP*	16.00
*CRHR1*	31.36	*APOE*	23.11	*GRIK3*	18.45	*HTT*	16.56	*PDSS1*	16.00
*AR*	30.79	*WFS1*	23.03	*AGTR1*	18.27	*CLOCK*	16.56	*GAL*	15.86
*DRD1*	30.63	*IL10*	22.88	*PDE1A*	18.24	*GRIN2B*	16.56	*AGT*	15.78
*PLXNA2*	30.09	*DRD2*	22.63	*CHRNA5*	18.20	*PCLO*	16.53	*TAC1*	15.75
*MAOA*	29.57	*DISC1*	22.59	*CPLX2*	18.20	*NRG1*	16.50	*NTRK2*	15.68
*OPRM1*	29.30	*PDE4B*	22.59	*HTR6*	18.15	*QKI*	16.47	*SLC6A1*	15.56
*IL6*	29.06	*CRHBP*	22.19	*CCK*	18.12	*GRIN2A*	16.42	*CTNNB1*	15.18
*HTR1B*	28.99	*PDE5A*	22.00	*ARRB2*	18.10	*DIO2*	16.42		

The proportion of genes expressed in 49 human tissues for 169 DEPgenes compared with 15,874 non-disease genes is shown in Supplementary Figure S3. Ten tissues exhibited expression differences greater than 4%. Among them, seven tissues were related to brain or nerve systems, including nervous (13.2%), brain (11.1%), peripheral nervous system (10.8%), cerebrum (9.2%), cerebellum (6.6%), eye (6%), and head and neck (4.2%), with the direction that the DEPgenes tended to express more in brain or nerve related tissues than non-disease genes.

## Discussion

A wealth of genetic data accumulated in the past decade regarding depression forms a special opportunity to uncover the biological functions and molecular mechanisms underlying depression through systematic data collection and integration. Our approach to prioritize genes according to their evidence in depression and using combined score to rank candidate genes for depression not only creates a value-added gene database for depression, but it also provides a list of candidates for future exploration of biological functions among these DEPgenes. A few existing databases have information on susceptible genes for depression by literature mining or by review of prior publications, such as HuGE navigator, to serve as a convenient searching engine. However, without a proper weighting scheme for the strength of evidence provided from different studies and data sources, these databases are less informative for follow-up studies. For instance, in HuGE Navigator (8 Feb 2011 version; http://www.hugenavigator.net/HuGENavigator/home.do), we searched gene information for depression and found 690 depression candidate genes with scores ranged between 0 and 1.5. Using a loose cutoff value of 0.01, we obtained 104 depression genes with their scores>0.01. There are 45 out of 104 HuGE depression genes not in our DEPgenes, with calculated mean combined score of 6.6 below our cutoff of 15. Some well-known depression candidate genes that do not have scores greater than 0.01 in the HuGE genes are included in our DEPgenes, such as *DBH*, *CHRNA7*, and *GABRA3*, which were all ranked in the top list of DEPgenes. Without proper evaluation of weighting scheme, using other search engines may result in omitting important information for follow-up studies.

The list of the prioritized DEPgenes can be used for individual replication and to further explore the biological roles of them in depression using basic science approaches. The top seven DEPgenes are *DBH*, *BDNF*, *SLC6A4*, *NGFR*, *TNF*, *GSK3B*, and *CHRNA7*. The roles of these high-ranking DEPgenes in depression were supported by review articles and empirical studies. For instance, increased dopaminergic activity may play a primary role in depression. Dopamine beta -hydroxylase (*DBH*) catalyses the key step in biosynthesis of the neurotransmitter noradrenaline from dopamine, and low *DBH* activity from a variety of brain regions is a possible risk factor for developing depression [32], [33]. Serotonin transporter (*SLC6A4*) and serotonin receptor (*HTR1A*, the 13^rd^) genes are among the strongest candidates underlying the etiology of depression [22], [34]. A commonly prescribed medication for treating depression is selective serotonin reuptake inhibitors (SSRIs) (paroxetine, fluoxetine, sertraline), which acts to keep the balance in the serotonin neurotransmitter system in the brain [35]. Brain-derived neurotrophic factor (*BDNF*) is a neuroprotective protein which alters the balance of neurotoxic and neuroprotective responses to stress by preventing hippocampal cells from damage and is suggested to be associated with depression [23], [36]. The nerve growth factor receptor (*NGFR*) encodes the affinity and modulates the activity of tyrosine kinases for neurotrophin family, and plays a potential role in ligand binding and signaling. The *NGFR* was reported to have protective effect against the development of depressive disorder [37]. The tumor necrosis factor (*TNF*) plays roles in altering neural-immune interactions, including levels of proinflammatory cytokines, increased pain sensitivity and elevated inflammatory activity [38]. Prior evidence supports that the development of depression is related to the levels of proinflammatory cytokines *TNF-α* and to interleukin-6 (*IL6*, the 33^rd^) in the brain [38]–[40]. Glycogen synthase kinase 3 beta (*GSK3B*) is an enzyme involved in energy metabolism and neuronal cell development, which are processes related to depression [36]. The *GSK3B* plays an important role in the action of mood stabilizer [41]. Lastly, the α7 neuronal nicotinic acetylcholine receptor subunit gene (*CHRNA7*) is a cholinergic receptor, which has been reported to be associated with a sensory deficit in common mental illness [42] and neurochemical changes in depression-like behavior [43]. Comparison of gene expression patterns of the DEPgenes with non-disease genes in human tissues exhibited high expression proportion among the DEPgenes in human brain or nerve related tissues. This is in accordance to the neurotransmitter action, which refers to the chemical message to influence intellectual functioning and behavior, and theories of neuroplasticity, which refers to the ability of learning to change through experience in human brain. Both expressions have been suggested to underlie the risk for depression [44].

Through comprehensive data collection, almost one-fourth of human genes were identified as susceptible genes for depression in one or several data sources. The candidate genes for depression across data sources had low overlap. This is partly reflected by poor replications across study designs and species in prior individual genetic studies. Several reasons may explain such observation, including heterogeneity of the depression phenotype, different study designs, lack of power in some studies, interaction of genetic and environmental factors, publication bias, and false-positive findings in most of the candidate gene studies [45].

The idea of using *preWeight* is to adjust for prior information/evidence imbalance across multidimensional data sources. If our results of genes ranking are robust, the list of DEPgenes should be similar with or without *preWeight* adjustment, and this is indeed what we observed. If *preWeight* was not applied, weight matrix [6,1,4,8,4,2,8] had the best performance and the corresponding prioritized genes set was very similar to those obtained using *preWeight* (data not shown). It is also worth noting that the weights for human and animal literature search were high regardless of using *preWeight* or not. This implicated that text-mining with efficient algorithm may exhibit a useful strategy to quickly discover the relationship between diseases and genes with less bias [19], [46].

The optimal weight matrix selection was based on two datasets in the current framework: a set of core genes through expert review and an independent GWA depression dataset. Previously suggested candidate genes from meta-analysis or review articles are still few, thus limiting the number of genes to be included in the core gene set. Having a representative core gene set of depression is essential to the final gene selection, as the numbers of weight matrices that satisfied the selection criteria were correlated with setting threshold of *ϕ* (proportion of core genes). Setting larger *ϕ* may assist to better identify an optimal weight matrix. It is possible that with an increasing number of core genes, we can allow the threshold to be lower. For the GWA dataset, although there were a few published GWA studies for depression [7], [9], [10], [17], only the GAIN dataset was deposited in a public repository and is freely available through an application process. If other GWA datasets could be acquired, the prioritization process can be cross validated by different GWA data to increase the precision and predictability in the current study, such that one GWA dataset can be used in random set comparison process and another GWA dataset can be used in p-value evaluation process, and so on. In sum, our selection of DEPgenes not only adopted proper weighting from multiple data sources, but also incorporated information from biological pathways. More exploratory and advanced pathway/network analyses can be conducted to further provide useful information from the created DEPgenes list. Similar data prioritization and evaluation procedures were used in other neuropsychiatric disorders, such as schizophrenia [19]. Sun et al., identified a list of schizophrenia candidate genes and successfully constructed pathways and networks among those genes [47]. Pathways overrepresented in their selected schizophrenia candidate genes were related to neurodevelopment and immune system. This is encouraging to conduct future work using system biological approach in the DEPgenes.

This study has some limitations. First, the choice of core genes was knowledge-based and subjective, which may influence the optimal weight matrix selection and the resulting DEPgenes. Nevertheless, our evaluations using different qualified weight matrices and alternative core gene sets found very similar list of DEPgenes with high correlation across weight matrices and comparable results from alternative pathway core gene set. Second, one may concern that larger genes were easier to be picked up by DEPgenes due to the bias of significant p-values towards gene length. In the GWA GAIN-MDD data, we observed a positive relationship between smaller p-values and larger genes among all human genes. However, there is no difference between the proportion of larger gene size (say >10000 kb) in the DEPgenes compared with other human genes (OR = 0.86, p-value = 0.47) and resulting random selected gene sets, which indicated that our selection of DEPgenes is unlikely impacted by the bias toward long gene length. Third, some of the candidate genes might be falsely reported in the literature as significant markers for depression and falsely collected as candidates, potentially providing incorrect evidence in our study. Similarly, while the phenotype of interest is depression, different studies may apply different measures and construct regarding “depression”, which may cause unavoidable noise in the evaluation process. Lastly, only human and available mouse data were considered in the current study. With increased data and knowledge accumulation in the near future, an updated and more precise DEPgenes list can be provided.

To our knowledge, this is the first comprehensive evidence-based candidate gene resource for depression. We expect the identification of potential susceptibility genes for depression will facilitate etiology and mechanism-related research. Through a systems biology view, new data generated by high-throughput genomics, proteomics or other relevant data sources could be utilized to extend the current dimensions of data collection, providing researchers an opportunity to implement pathway- or network-based analysis to explore the underlying functional correlation among susceptible genes of depression in the near future.

## Supporting Information

Figure S1 **Distributions of the GWA** ***p*** **-values (GAIN) of prioritized genes corresponding to ten selected weight matrices.**
(DOC)

Figure S2 **Distributions of combined scores in the core genes and all candidate genes.**
(DOC)

Figure S3 **Distributions of proportion of gene expression in 49 human tissues between prioritized genes (DEPgenes) and non-disease genes.**
(DOC)

Table S1 **The score scheme of candidate genes from different data sources.**
(DOC)

Table S2 **A list of KEGG pathways related to monoamine-deficiency hypothesis, hypothalamic pituitary adrenal axis, and other possible pathophysiological mechanisms for depression.**
(DOC)

Table S3 **Distribution of depression candidate genes in seven data sources.**
(DOC)

Table S4 **The Spearman's correlation coefficient of ranked prioritized gene sets derived from the ten selected weight matrices.**
(DOC)

Text S1 **Weight matrix selection and the Selection criteria of optimal weight matrix.**
(DOC)

Text S2 **Robustness test.**
(DOC)

Text S3 **Using the best expression and pathway genes as core gene sets.**
(DOC)

## References

[1] Harvey M, Belleau P, Barden N (2007). Gene interactions in depression: pathways out of darkness.. TRENDS in Genetics.

[2] Bijl RV, Ravelli A, van Zessen G (1998). Prevalence of psychiatric disorder in the general population: Results of The Netherlands Mental Health Survey and Incidence Study (NEMESIS).. Social Psychiatry Psychiatric Epidemiology.

[3] Kessler RC, Berglund P, Demler O, Jin R, Merikangas KR (2005). Lifetime prevalence and age-of-onset distributions of DSM-IV disorders in the National Comorbidity Survey Replication.. Archives of General Psychiatry.

[4] Vicente B, Kohn R, Rioseco P, Saldivia S, Levav I (2006). Lifetime and 12-month prevalence of DSM-III-R disorders in the Chile psychiatric prevalence study.. American Journal of Psychiatry.

[5] Sullivan PF, Neale MC, Kendler KS (2000). Genetic epidemiology of major depression: review and meta-analysis.. American Journal of Psychiatry.

[6] Kuo P-H, Neale MC, Riley BP, Patterson DG, Walsh D (2007). A genome-wide linkage analysis for the personality trait neuroticism in the Irish affected sib-pair study of alcohol dependence.. Neuropsychiatric Genetics.

[7] Muglia P, Tozzi F, Galwey N, Francks C, Upmanyu R (2010). Genome-wide association study of recurrent major depressive disorder in two European case-control cohorts.. Molecular Psychiatry.

[8] Nash MW, Huezo-Diaz P, Williamson RJ, Sterne A, Purcell S (2004). Genome-wide linkage analysis of a composite index of neuroticism and mood-related scales in extreme selected sibships.. Human Molecular Genetics.

[9] Shi J, Potash J, Knowles J, Weissman M, Coryell W (2010). Genome-wide association study of recurrent early-onset major depressive disorder.. Molecular Psychiatry.

[10] Sullivan PF, Geus EJCd, Willemsen G, James MR, Smit JH (2009). Genome-wide association for major depressive disorder: a possible role for the presynaptic protein piccolo.. Molecular Psychiatry.

[11] Uriguen L, Arteta D, Diez-Alarcia R, Ferrer-Alcon M, Diaz A (2008). Gene expression patterns in brain cortex of three different animal models of depression.. Genes, Brain and Behavior.

[12] Wray NR, Middeldorp CM, Birley AJ, Gordon SD, Sullivan PF (2008). : Genome-wide linkage analysis of multiple measures of neuroticism of 2 large cohorts from Australia and the Netherlands.. Archives of General Psychiatry.

[13] Belmaker RH, Agam G (2008). Major depressive disorder.. New England Journal of Medicine.

[14] Krishnan V, Nestler EJ (2008). The molecular neurobiology of depression.. Nature.

[15] MacMaster FP, Russell A, Mirza Y, Keshavan MS, Taormina SP (2006). Pituitary volume in treatment-naïve pediatric major depressive disorder.. Biological Psychiatry.

[16] Merali Z, Du L, Hrdina P, Palkovits M, Faludi G (2004). Dysregulation in the Suicide Brain: mRNA Expression of Corticotropin-Releasing Hormone Receptors and GABAA Receptor Subunits in Frontal Cortical Brain Region.. The Journal of Neuroscience.

[17] Shyn S, Shi2 J, Kraft J, Potash J, Knowles J (2009). Novel loci for major depression identified by genome-wide association study of Sequenced Treatment Alternatives to Relieve Depression and meta-analysis of three studies.. Molecular Psychiatry.

[18] Ma X, Lee H, Wang L, Sun F (2007). CGI: a new approach for prioritizing genes by combining gene expression and protein-protein interaction data.. Bioinformatics.

[19] Sun J, Jia P, H.Fanous A, Webb BT, Oord EJCGvd (2009). A multi-dimensional evidence-based candidate gene prioritization approach for complex diseases-schizophrenia as a case.. Bioinformatics.

[20] Guo A-Y, Webb BT, Miles MF, Zimmerman MP, Kendler KS (2009). ERGR: An ethanol-related gene resource.. Nucleic Acids Research.

[21] Rankinen T, Zuberi A, Chagnon YC, Weisnagel SJ, Argyropoulos G (2006). The Human Obesity Gene Map: The 2005 Update.. Obesity.

[22] Kato M, Serretti A (2010). Review and meta-analysis of antidepressant pharmacogenetic findings in major depressive disorder.. Molecular Psychiatry.

[23] Levinson DF (2006). The genetics of depression: a review.. Biological Psychiatry.

[24] Wray N, Pergadia M, Blackwood D, Penninx B, Gordon S (2010). Genome-wide association study of major depressive disorder: new results, meta-analysis, and lessons learned.. Molecular Psychiatry.

[25] López-León S, Janssens ACJW, Ladd AMG-Z, Del-Favero J, Claes SJ (2008). Meta-analyses of genetic studies on major depressive disorder.. Molecular Psychiatry.

[26] Higgs BW, Elashoff M, Richman S, Barci B (2006). An online database for brain disease research.. BMC Genomics.

[27] Hunsberger JG, Newton SS, Bennett AH, Duman CH, Russell DS (2007). : Antidepressant actions of the exercise-regulated gene VGF.. Nature Medicine.

[28] Kato T (2007). Molecular genetics of bipolar disorder and depression.. Psychiatry and Clinical Neurosciences.

[29] Kanehisa M, Goto S (2000). KEGG: Kyoto Encyclopedia of Genes and Genomes.. Nucleic Acids Research.

[30] Kanehisa M, Goto S, Furumichi M, Tanabe M, Hirakawa M (2010). KEGG for representation and analysis of molecular networks involving diseases and drugs.. Nucleic Acids Research.

[31] Zhang B, Kirov S, Snoddy J (2005). WebGestalt: an integrated system for exploring gene sets in various biological contexts.. Nucleic Acids Research.

[32] Wood JG, Joyce PR, Miller AL, Mulder RT, Kennedy MA (2002). A polymorphism in the dopamine β-hydroxylase gene is associated with “Paranoid Ideation” in patients with major depression.. Biological Psychiatry.

[33] Cubells JF, Zabetian CP (2004). Human genetics of plasma dopamine β-hydroxylase activity: applications to research in psychiatry and neurology.. Psychopharmacology.

[34] Pezawas L, Meyer-Lindenberg A, Drabant EM, Verchinski BA, Munoz KE (2005). 5-HTTLPR polymorphism impacts human cingulate-amygdala interactions: a genetic susceptibility mechanism for depression.. Nature Neuroscience.

[35] Nurnberg HG, Thompson PM, Hensley PL (1999). Antidepressant medication change in a clinical treatment setting: a comparison of the effectiveness of selective serotonin reuptake inhibitors.. Journal of Clinical Psychiatry.

[36] Zhang K, Yang C, Xu Y, Sun N, Yang H (2010). Genetic association of the interaction between the BDNF and GSK3B genes and major depressive disorder in a Chinese population.. Journal of Neural Transmission.

[37] Kunugi H, Hashimoto R, Yoshida M, Tatsumi M, Kamijima K (2004). A missense polymorphism (S205L) of the low-affinity neurotrophin receptor p75NTR gene Is associated with depressive disorder and attempted suicide.. American Journal of Medical Genetics Part B (Neuropsychiatric Genetics).

[38] Euteneuer F, Schwarz MJ, Hennings A, Riemer S, Stapf T (2010). Depression, cytokines and experimental pain: Evidence for sex-related association patterns.. Journal of Affective Disorders.

[39] Ertenli I, Ozer S, Kiraz S, Apras SB, Akdogan A (2010). Infliximab, a TNF-alpha antagonist treatment in patients with ankylosing spondylitis: the impact on depression, anxiety and quality of life level.. Rheumatology International.

[40] Dowlati Y, Herrmann N, Swardfager W, Liu H, Sham L (2010). A meta-analysis of cytokines in major depression.. Biological Psychiatry.

[41] Jope RS, Bijur GN (2002). Mood stabilizers, glycogen synthase kinase-3B and cell survival.. Molecular Psychiatry.

[42] Leonard S, Gault J, Hopkins J, Logel J, Vianzon R (2002). Association of promoter variants in the 7 nicotinic acetylcholine receptor subunit gene with an inhibitory deficit found in schizophrenia.. Archives of General Psychiatry.

[43] Blaveri E, Kelly F, Mallei A, Harris K, Taylor A (2010). Expression profiling of a genetic animal model of depression reveals novel molecular pathways underlying depressive-like behaviours.. PLoS ONE.

[44] Schildkraut JJ (1965). The catecholamine hypothesis of affective disorders: A review of supporting evidence.. American Journal of Psychiatry.

[45] Bosker F, Hartman C, Nolte I, Prins B, Terpstra P (2010). Poor replication of candidate genes for major depressive disorder using genome-wide association data.. Molecular Psychiatry.

[46] Tiffin N, Kelso JF, Powell AR, Pan H, Bajic VB (2005). Integration of text- and data-mining using ontologies successfully selects disease gene candidates.. Nucleic Acids Research.

[47] Sun J, Jia P, Fanous AH, Oord Evd, Chen X (2010). Schizophrenia gene networks and pathways and their applications for novel candidate gene selection.. PLoS ONE.

