# Supplementary material for: Prioritization and Evaluation of Depression Candidate Genes by Combining Multidimensional Data Resources
Source: PLoS One. 2011 Apr 6;6(4):e18696. doi: 10.1371/journal.pone.0018696 (PMC3071871; doi:10.1371/journal.pone.0018696)
Supplement: Figure S1 — Distributions of the GWA p -values (GAIN) of prioritized genes corresponding to ten selected weight matrices. (DOC) [file pone.0018696.s001.doc]

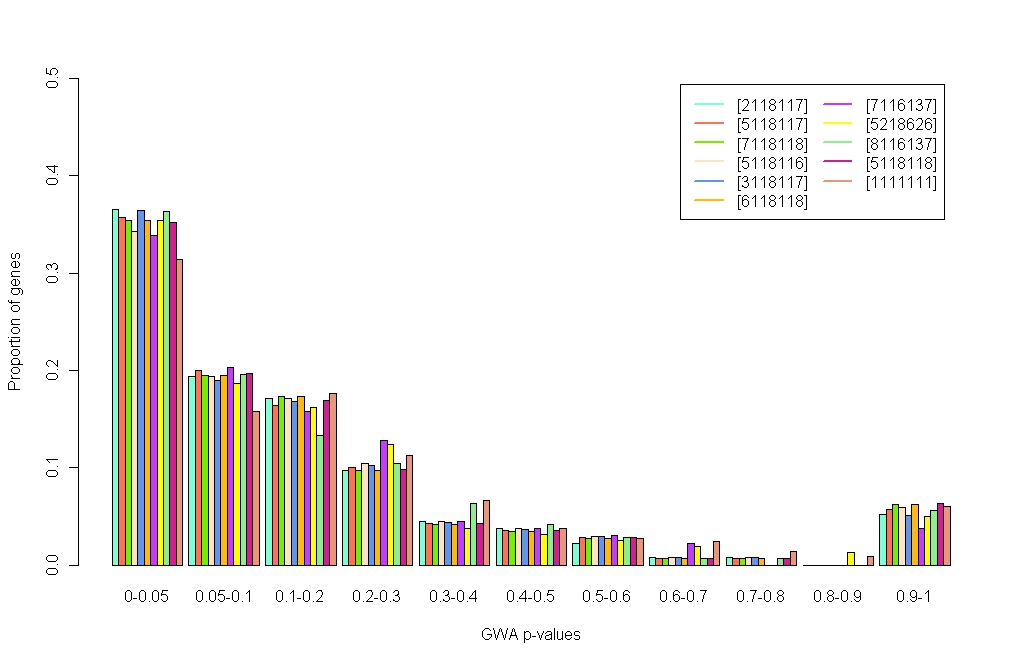


Figure S1. Distributions of the GWA *p*-values (GAIN) of prioritized genes corresponding to ten selected weight matrices.
