# Supplementary figures and images for: Prioritization and Evaluation of Depression Candidate Genes by Combining Multidimensional Data Resources
Source: PLoS One. 2011 Apr 6;6(4):e18696. doi: 10.1371/journal.pone.0018696 (PMC3071871; doi:10.1371/journal.pone.0018696)

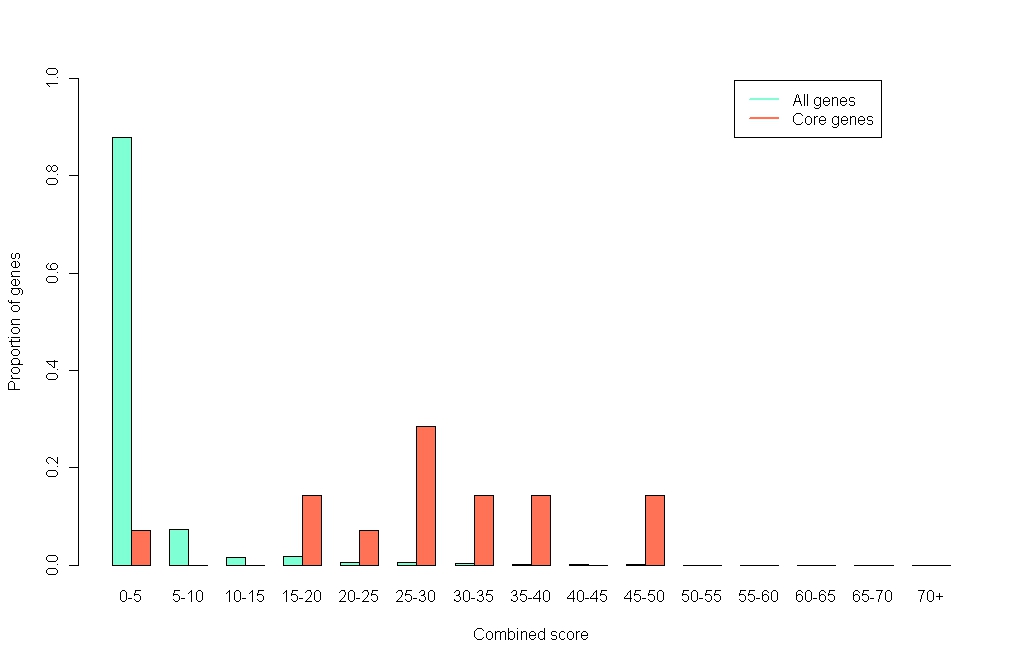


Figure S2. Distributions of combined scores in the core genes and all candidate genes.

Supplement: Figure S2 — Distributions of combined scores in the core genes and all candidate genes. (DOC) [file pone.0018696.s002.doc]
