# Supplementary material for: Prioritization and Evaluation of Depression Candidate Genes by Combining Multidimensional Data Resources
Source: PLoS One. 2011 Apr 6;6(4):e18696. doi: 10.1371/journal.pone.0018696 (PMC3071871; doi:10.1371/journal.pone.0018696)
Supplement: Figure S3 — Distributions of proportion of gene expression in 49 human tissues between prioritized genes (DEPgenes) and non-disease genes. (DOC) [file pone.0018696.s003.doc]

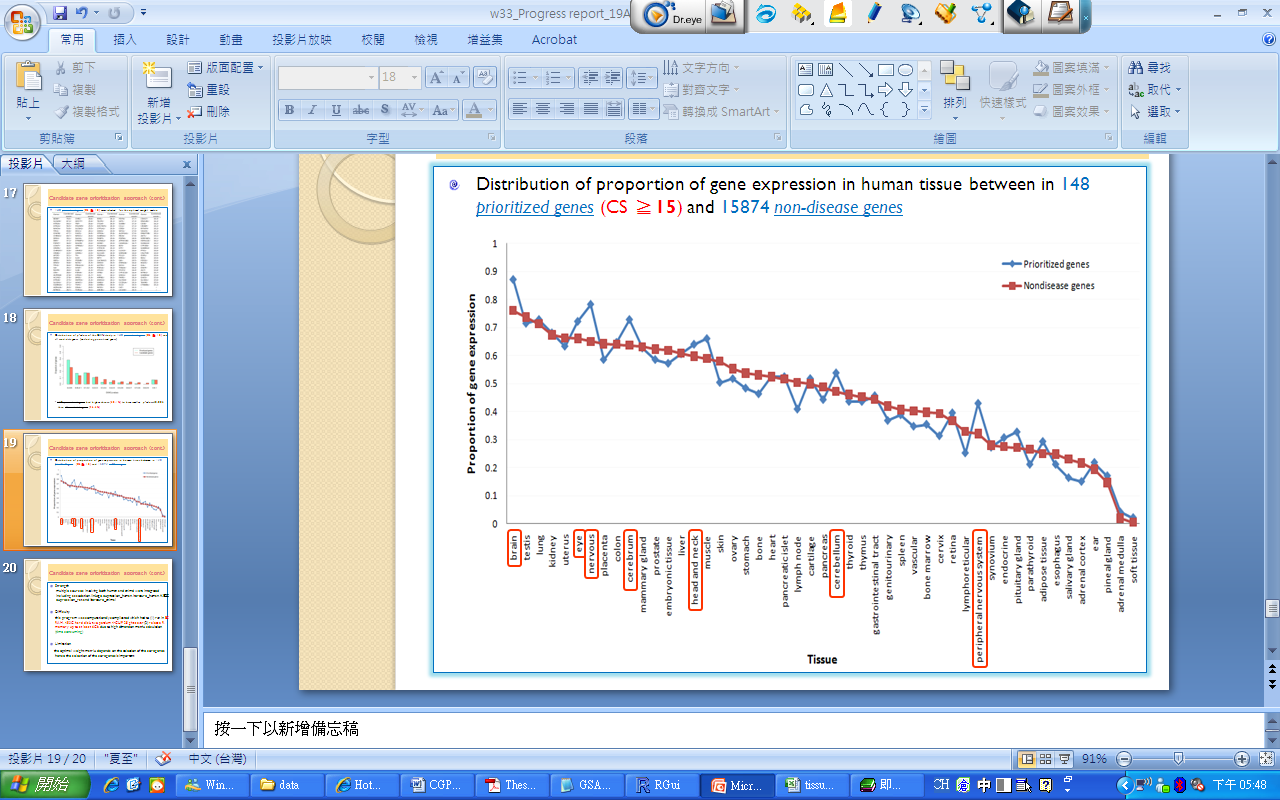


Figure S3. Distributions of proportion of gene expressed in 49 human tissues between prioritized genes (DEPgenes) and non-disease genes.
