# Supplementary material for: Prioritization and Evaluation of Depression Candidate Genes by Combining Multidimensional Data Resources
Source: PLoS One. 2011 Apr 6;6(4):e18696. doi: 10.1371/journal.pone.0018696 (PMC3071871; doi:10.1371/journal.pone.0018696)
Supplement: Table S1 — The score scheme of candidate genes from different data sources. (DOC) [file pone.0018696.s004.doc]

Table S1. The score scheme of candidate genes from different data sources

| Data source | Score criteria | Score range | *preWeight* |
| --- | --- | --- | --- |
| Association | 4 (*pp*>0.75 & *total*>3)  3 (0.5<*pp*≤0.75 & *total*>3) or  (*pp=1 & total*≤3)  2 (*pp*≤0.5 & *total>3*) or (*pp≥0.5 & total≤3*)  1 (*pos*=1)  0 (*pp*=0) | [0, 4] | 1.5 |
| Linkage | 4 (4≤LOD)  3 (3≤LOD<4)  2 (2≤LOD<3)  1 (1≤LOD<2)  0 (LOD<1)  Max(LOD, -log(*p*-value)) | [0, 4.6] | 1.0 |
| Expression (human) | -log(*p*-value) | [0. 4.6] | 1.5 |
| Literature (human) | Number of hits | [1, 6] | 0.5 |
| Regulatory pathway | 3 (monoamine deficiency hypothesis)  2 (hypothalamic pituitary adrenal axis)  1 (other possible mechanisms) | [0, 5.6] | 1.0 |
| Expression (animal) | -log(*p*-value) | [0, 5.6] | 1.0 |
| Literature (animal) | Number of hits | [1, 4] | 0.5 |

Note: *pp* represents proportion of positive results; *pos* represents positive results; and *total* represents total number of studies. A *preWeight* (0.5 to 1.5) was defined as a pre-weighting scheme to the seven data categories to adjust for varying score ranges across data sources originally.
