# Supplementary material for: Prioritization and Evaluation of Depression Candidate Genes by Combining Multidimensional Data Resources
Source: PLoS One. 2011 Apr 6;6(4):e18696. doi: 10.1371/journal.pone.0018696 (PMC3071871; doi:10.1371/journal.pone.0018696)
Supplement: Table S2 — A list of KEGG pathways related to monoamine-deficiency hypothesis, hypothalamic pituitary adrenal axis, and other possible pathophysiological mechanisms for depression. (DOC) [file pone.0018696.s005.doc]

Table S2. A list of KEGG pathways related to monoamine-deficiency hypothesis, hypothalamic pituitary adrenal axis, and other possible pathophysiological mechanisms for depression

| Hypothesis | Molecule | KEGG pathway |
| --- | --- | --- |
| Monoamine-deficiency hypothesis | Dopamine | Tyrosine metabolism (hsa00350) |
|  | Serotonin | Glycine, serine and threonine metabolism (hsa00260)  Tryptophan metabolism (hsa00380) |
| Hypothalamic pituitary adrenal axis | Vasopressin | Calcium signaling pathway (hsa04020)  Vascular smooth muscle contraction (hsa04270) |
|  | Cytokine | Cytokine-cytokine receptor interaction (hsa04060)  Chemokine signaling pathway (hsa04062) |
| Other possible pathophysiological mechanisms | Glutamatergic neurotransmission | D-Glutamine and D-glutamate metabolism (hsa00471) |
|  | GABAergic neurotransmission | Regulation of autophagy (hsa04140) |
|  | Circadian rhythm | Circadian rhythm - mammal (hsa04710) |
|  | Neurosteroid synthesis | Steroid hormone biosynthesis (hsa00140) |
|  | Acetylcholine | Glycerophospholipid metabolism (hsa00564) |
