# Supplementary material for: Prioritization and Evaluation of Depression Candidate Genes by Combining Multidimensional Data Resources
Source: PLoS One. 2011 Apr 6;6(4):e18696. doi: 10.1371/journal.pone.0018696 (PMC3071871; doi:10.1371/journal.pone.0018696)
Supplement: Table S3 — Distribution of depression candidate genes in seven data sources. (DOC) [file pone.0018696.s006.doc]

Table S3. Distribution of depression candidate genes in seven data sources

| Data source | Number of genes | Number of genes overlapped | | | | | |
| --- | --- | --- | --- | --- | --- | --- | --- |
|  |  | Association | Linkage | Expression  (human) | Literature  (human) | Pathway | Expression  (animal) |
| Association | 125 |  |  |  |  |  |  |
| Linkage | 3628 | 12 ( 0.3%) |  |  |  |  |  |
| Expression (human) | 301 | 57(18.9%) | 43 (14.3%) |  |  |  |  |
| Literature (human) | 473 | 60 (12.7%) | 93 (19.7%) | 9 (1.9%) |  |  |  |
| Pathway | 827 | 23 ( 2.8%) | 123 (14.9%) | 28 (3.4%) | 120 (14.5%) |  |  |
| Expression (animal) | 252 | 3 ( 1.2%) | 39 (15.5%) | 9 (3.6%) | 22 ( 8.7%) | 31 (12.3%) |  |
| Literature (animal) | 306 | 22 ( 7.2%) | 20 ( 6.5%) | 5 (1.6%) | 76 (24.8%) | 41 (13.4%) | 5 (1.6%) |
