# Supplementary material for: Prioritization and Evaluation of Depression Candidate Genes by Combining Multidimensional Data Resources
Source: PLoS One. 2011 Apr 6;6(4):e18696. doi: 10.1371/journal.pone.0018696 (PMC3071871; doi:10.1371/journal.pone.0018696)
Supplement: Table S4 — The Spearman's correlation coefficient of ranked prioritized gene sets derived from the ten selected weight matrices. (DOC) [file pone.0018696.s007.doc]

Table S4. The Spearman’s correlation coefficient of ranked prioritized gene sets derived from the ten selected weight matrices

| Weight  matrix | Weight matrix | | | | | | | | | |
| --- | --- | --- | --- | --- | --- | --- | --- | --- | --- | --- |
|  | [2118117] | [5118117] | [7118118] | [5118116] | [3118117] | [6118118] | [7116137] | [5218626] | [8116137] | [5118118] |
| [2118117] | 1 (169) | 0.87(169) | 0.84(169) | 0.85(168) | 0.94(169) | 0.85(169) | 0.51(110) | 0.84(168) | 0.51(110) | 0.87(169) |
| [5118117] |  | 1 (177) | 0.98(177) | 0.99(176) | 0.97(172) | 0.99(177) | 0.97(118) | 0.94(176) | 0.97(118) | 1.00(177) |
| [7118118] |  |  | 1 (178) | 0.97(176) | 0.95(172) | 1.00(178) | 0.99(118) | 0.92(177) | 0.99(118) | 0.98(178) |
| [5118116] |  |  |  | 1 (176) | 0.96(171) | 0.98(176) | 0.97(118) | 0.93(176) | 0.97(118) | 0.99(178) |
| [3118117] |  |  |  |  | 1 (172) | 0.96(172) | 0.84(113) | 0.92(171) | 0.83(113) | 0.97(172) |
| [6118118] |  |  |  |  |  | 1 (178) | 0.98(118) | 0.93(177) | 0.98(118) | 0.99(178) |
| [7116137] |  |  |  |  |  |  | 1 (128) | 0.88(126) | 1.00(128) | 0.96(118) |
| [5218626] |  |  |  |  |  |  |  | 1 (437) | 0.87(126) | 0.94(177) |
| [8116137] |  |  |  |  |  |  |  |  | 1 (128) | 0.96(118) |
| [5118118] |  |  |  |  |  |  |  |  |  | 1 (178) |

Note: (1) All correlation is statistically significant at 0.001 level (2-tailed).

(2) The number in brackets represents the number of overlapping genes between the two gene sets.
