# Supplementary material for: Prioritization and Evaluation of Depression Candidate Genes by Combining Multidimensional Data Resources
Source: PLoS One. 2011 Apr 6;6(4):e18696. doi: 10.1371/journal.pone.0018696 (PMC3071871; doi:10.1371/journal.pone.0018696)
Supplement: Text S1 — Weight matrix selection and the Selection criteria of optimal weight matrix. (DOC) [file pone.0018696.s008.doc]

**Text S1. Weight matrix selection and the Selection criteria of optimal weight matrix**

In the weight matrix selection step, two parameters, *φ* (proportion of core genes) and *η* (proportion of candidate genes) were then introduced to assure that *φ* of the core genes being ranked in the top list of *η* of sorted candidate genes. The position *j* denoted as the position of the *φ*-th core gene locates in the candidate gene list and was used to select weight matrix. We considered nine combinations of threshold values with parameters *φ*=0.9 (13 core genes), 0.85 (12 core genes) and 0.8 (11 core genes), and *η*= 0.05 (top 253 genes), 0.04 (top 202 genes) and 0.03 (top 152 genes). The weight matrices that fulfill these threshold criteria were retained for the next evaluation step.

Selection criteria of optimal weight matrix is based on a lower position *j* (≤160), position *l* (≤1200, the position of the last core gene in the ranked candidate gene list), a greater mean (≥900, total number of the random sets having significant larger *p*-value distribution than the prioritized set), and a smaller standard deviation.
