# Supplementary material for: Prioritization and Evaluation of Depression Candidate Genes by Combining Multidimensional Data Resources
Source: PLoS One. 2011 Apr 6;6(4):e18696. doi: 10.1371/journal.pone.0018696 (PMC3071871; doi:10.1371/journal.pone.0018696)
Supplement: Text S2 — Robustness test. (DOC) [file pone.0018696.s009.doc]

**Text S2. Robustness test**

We applied two approaches to test the robustness of choosing specific weight matrix as the optimal one on the selection of DEPgenes. First, we selected ten weight matrices ([2,1,1,8,1,1,7], [5,1,1,8,1,1,7], [7,1,1,8,1,1,8], [3,1,1,8,1,1,7], [6,1,1,8,1,1,8], [5,1,1,8,1,1,8], [5,1,1,8,1,1,6], [7,1,1,6,1,3,7], [5,2,1,8,6,2,6], and [8,1,1,6,1,3,7]) that passed selection criteria to evaluate their performance using the GWA dataset. The top ranked prioritized gene set choosing by the pre-defined cutoff value was obtained for each of the ten matrixes, and the corresponding distribution of the GWA *p*-values of each of the ten prioritized gene set was plotted (see Supplementary Figure S1). In addition, to investigate whether the rank of prioritized genes obtaining from each weight matrix were similar, pair-wise comparisons for the ranks of prioritized genes among ten matrices (in total 45 pairs of prioritized gene set comparisons) were calculated using Spearman’s correlation coefficients. A high on average correlation in these comparisons would demonstrate the effectiveness and robustness of this prioritization approach.
