# Supplementary material for: Prioritization and Evaluation of Depression Candidate Genes by Combining Multidimensional Data Resources
Source: PLoS One. 2011 Apr 6;6(4):e18696. doi: 10.1371/journal.pone.0018696 (PMC3071871; doi:10.1371/journal.pone.0018696)
Supplement: Text S3 — Using the best expression and pathway genes as core gene sets. (DOC) [file pone.0018696.s010.doc]

**Text S3. Using the best expression and pathway genes as core gene sets**

To determine the best expression genes, we used criteria as follow: (1) selected top 10 genes separately from human and from animal studies if its p-value <0.01 and fold change>1.2; and (2) selected overlapping genes from human and animal studies for genes with p-value<0.05 and fold change >1.2. As a result, there were 20 best expression genes identified. We used these 20 genes as the core genes to find the best matrices. However, we found no any best matrices meet selection criteria using the same parameters.

The best candidate pathway genes are more difficult to define. Because depression is well-known related to serotonin, we take serotonin related pathways as example – using genes from Glycine, serine and threonine metabolism (hsa00260; 32 genes) and tryptophan metabolism (hsa00380; 42 genes) pathways. As a result, 17 genes whose p-values are less than 0.05 were identified as the pathway core genes. An optimal matrix [6,2,1,8,7,1,8] was obtained for depression based on the pathway core gene set and resulted in 114 pathway-DEPgenes.
